# Declines in HIV testing and diagnoses: a policy analysis of the 2019 Title X federal regulations on family planning clinics

**DOI:** 10.1080/26410397.2026.2670146

**Published:** 2026-05-08

**Authors:** Jennifer Sherwood, Nathan Roberson, Deborah Stenoien, Elise Lankiewicz, Brian Honermann, Greg Millett

**Affiliations:** a Director of Research, Public Policy, amfAR The Foundation for AIDS Research, Washington, DC, USA.; b Consultant, Public Policy Office, amfAR The Foundation for AIDS Research, Washington, DC, USA; c Public Policy Fellow, Public Policy Office, amfAR The Foundation for AIDS Research, Washington, DC, USA; d Policy Associate, Public Policy Office, amfAR The Foundation for AIDS Research, Washington, DC, USA; e Deputy Director, Public Policy Office, amfAR The Foundation for AIDS Research, Washington, DC, USA; f Vice President and Director, Public Policy Office, amfAR The Foundation for AIDS Research, Washington DC, USA

**Keywords:** HIV, family planning, sexual and reproductive health policy, Title X

## Abstract

In 2019 the Trump administration instituted a federal regulation (hereinafter the Policy) requiring that Title X family planning clinics have financial and physical separation of abortion services from other health services, and prohibited abortion referrals. In response, 32% (1,280) of Title X funded sites left the programme nationally. Our analysis connects public data from Title X Family Planning Reports (2016–2021) and CDC surveillance reports (2016–2021), to examine the Policy’s impact on: (1) HIV testing at Title X clinics by region, and (2) the proportion of a region’s total HIV diagnoses at Title X clinics. We use mixed multivariable linear regression modelling to examine interactions between “high” regional-level Policy exposure (defined as >25% Policy-related clinic withdrawal from the programme) and pre- and post-Policy implementation for HIV outcomes. Interaction models showed that while in effect the Policy resulted in 69,626 fewer HIV tests (95% CI −108,893 to −30,359 *P* < .001) and a 4% reduction (95% CI −7% to −0%; *P* = .052) in overall regional HIV diagnoses at Title X clinics in high-exposed regions compared to low-exposed regions. Results show notable declines in HIV testing and the proportion of HIV cases diagnosed at Title X sites as a result of Policy implementation. Policies that endanger the Title X family planning network also weaken the US HIV response. Future policies governing the Title X family planning programme should consider the full consequences for sexual and reproductive health outcomes, including HIV, in the US before implementation.

## Plain language summary

The federally funded Title X Family Planning Programme is a key provider of free and reduced cost sexual and reproductive health services, including HIV prevention, in the United States (US). The programme is especially important for low-income women who may face barriers at other facilities. We must examine any policies governing this safety net programme to ensure that these populations still get the best care. In 2019 the Trump administration passed a federal rule affecting clinics receiving Title X funding by requiring complete separation of abortion services from other health services. This rule (the Policy) also prohibited clinics from making abortion referrals for their patients. About a third of clinics in the Title X programme across the US refused to comply with these restrictions and gave up federal Title X funding. Title X clinics are very important for HIV testing services. We examined the impact of this mass clinic withdrawal on HIV testing services and HIV diagnoses in the Title X programme. We found that there were significant reductions related to the Policy in both HIV testing and diagnoses in the Title X programme. Our results show how policies that reduce providers in the Title X family planning network also weaken the US HIV response. Sound health policy that advances efforts to end the US HIV epidemic will support reproductive health clinics, such as those in the Title X programme, as key providers of HIV services.

## Résumé

En 2019, l’administration Trump a instauré une réglementation fédérale (ci-après la “ politique ”) exigeant que les centres de planification familiale relevant du titre X assurent une séparation financière et matérielle des services d'avortement et des autres services de santé, et interdisant l'orientation des patientes vers l'avortement. En conséquence, 32% (1280) des sites financés par le titre X ont quitté le programme à l'échelle nationale. Notre analyse croise les données publiques tirées des rapports sur la planification familiale du titre X (2016–2021) et des rapports de surveillance des CDC (2016-2021) afin d'examiner les répercussions de la politique sur : (1) le dépistage du VIH dans les centres relevant du titre X par région; et (2) la proportion des diagnostics du VIH dans les centres du titre X au sein d'une région. Nous avons utilisé un modèle de régression linéaire multivariée mixte pour examiner les interactions entre une exposition “ élevée ” à la politique au niveau régional (définie comme un retrait du programme par le centre en lien avec la politique supérieur à 25%) et les résultats en matière de VIH avant et après la mise en œuvre de la politique. Les modèles d'interaction ont montré que la politique a effectivement entraîné une diminution de 69 626 tests de dépistage du VIH (IC 95%, −108 893 à −30 359; *p *< 0,001) et une réduction de 4% (IC 95%, −7% à −0%; *p* = 0,052) du nombre total de diagnostics du VIH dans les centres du titre X des régions à forte exposition, par comparaison aux régions à faible exposition. Les résultats révèlent des baisses sensibles du dépistage du VIH et de la proportion de cas diagnostiqués dans les sites du titre X suite à l'application de cette politique. Les politiques qui mettent en danger le réseau de planification familiale du titre X affaiblissent également la riposte des États-Unis au VIH. Les futures politiques régissant le programme de planification familiale du titre X doivent tenir compte de l'ensemble des conséquences sur la santé sexuelle et reproductive, notamment en matière de VIH, aux États-Unis, avant leur mise en œuvre.

## Resumen

En 2019 el gobierno de Trump instituyó un reglamento federal (en adelante la Política) que exige que las clínicas de planificación familiar del Título X mantengan separación financiera y física entre los servicios de aborto y otros servicios de salud, y se les prohíbe hacer referencias a servicios de aborto. En respuesta, el 32% (1280) de los sitios financiados por el Título X dejaron el programa a nivel nacional. Nuestro análisis conecta los datos públicos de los Informes de planificación familiar del Título X (2016-2021) y los informes de vigilancia de los CDC (2016–2021), para examinar el impacto de la Política en: (1) pruebas de VIH en clínicas del Título X por región; y (2) el porcentaje del total de diagnósticos de VIH de cada región en las clínicas del Título X. Utilizamos modelos de regresión lineal multivariable mixtos para examinar las interacciones entre “alta” exposición a la Política a nivel regional (definida como >25% de retiro de la clínica del programa a causa de la Política) y antes y después de la aplicación de la Política para los resultados de VIH. Los modelos de interacción mostraron que mientras que la Política estuvo vigente, se realizaron 69,626 menos pruebas de VIH (IC de 95% −108,893 a −30,359 *P *< .001) y hubo una reducción del 4% (IC de 95% −7% a −0%; *P* = .052) en los diagnósticos de VIH generales en las clínicas del Título X en regiones de alta exposición comparadas con las regiones de baja exposición. Los resultados muestran disminuciones notables en las pruebas de VIH y en el porcentaje de casos de VIH diagnosticados en los sitios del Título X como consecuencia de la aplicación de la Política. Las políticas que ponen en peligro la red de planificación familiar del Título X también debilitan la respuesta de Estados Unidos al VIH. Futuras políticas que rijan el programa de planificación familiar del Título X deben considerar todas las consecuencias para los resultados en salud sexual y reproductiva, incluido el VIH, en Estados Unidos antes de su aplicación.

## Introduction

The Office of Population Affairs’ Title X Family Planning Programme (Title X) is the only federal programme in the United States (US) dedicated solely to providing comprehensive family planning and related preventive health services. Established in 1970, the programme aims to ensure equitable access to contraception, sexually transmitted infection (STI) and HIV testing, cancer screenings, and pregnancy counselling in the US. Federal Title X grants are competitively awarded to state health departments, non-profit organisations, and community health centres, which in turn deliver services through thousands of clinical sites nationwide. Title X primarily serves individuals with low incomes or without health insurance, playing a critical role in reducing barriers to sexual and reproductive health care in the US, where access can vary widely depending on a person’s state of residence and insurance status.^1^ Historically, the Title X programme has supported between 3,000 and 4,000 clinics nationally depending on the year.^2^ However, US policy changes to Title X funding and regulations have had a substantial impact on these numbers over time.

In March 2019, the Department of Health and Human Services (HHS) under the Trump Administration instituted a regulation (hereinafter “the Policy”) requiring financial and physical separation between Title X funded programmes and facilities where abortion is provided.^3^ The 2019 Policy further specified that Title X funded providers were mandated to refer pregnant clients for prenatal care and could not refer clients for abortion services.^4^ In response, 32% (1,280) of the nation’s Title X funded sites refused to comply with the Policy and left the programme between June 2019 and August 2021.^5,6^

This mass withdrawal of Title X sites had a significant impact on the programme’s family planning and STI service delivery, and hence on the sexual and reproductive rights of users. In 2019, Title X funded clinics served 844,000 fewer family planning users than in 2018.^7^ The 2019 Family Planning Annual Report by the Office of Population Affairs, cites the change in Title X programme regulations as a key reason for the reduced capacity and performance of the service network.^7^ In 2020, the effects of the Policy were compounded by the COVID-19 pandemic, and Title X funded clinics served 2.4 million fewer family planning users than in 2018.^8^ Of this total reduction, an estimated 63% was attributed to the Policy, while 37% was attributed to the COVID-19 pandemic.^8^ Similarly, Policy-related clinic loss was associated with significant reductions in chlamydia, gonorrhoea, and syphilis testing at Title X clinics beyond declines related to the COVID pandemic.^9^ The effects of the Policy on HIV services have not been widely reported. Examining the effect of the Policy on HIV testing and diagnoses is an important research area, given the historical contribution of Title X sites to the US HIV testing infrastructure and the potential for Policy-related disruptions to the US HIV response.^10^

In November 2021, the Biden Administration finalised a new set of Title X regulations to reverse those of the previous Trump Administration. As a result, clinics began to rejoin the Title X programme. By May 2023, the total Title X network had 2% more sites than prior to the Trump era regulations.^6,10^ However, this progress is fragile. Several actions by the second Trump Administration in 2025 indicate an intention to restrict funding for certain Title X providers or eliminate the programme entirely. In March 2025, the Administration announced a freeze on Title X funds to 16 of the 86 grantees – organisations that collectively supported 879 health centres across 23 states.^11^ Service delivery at these sites is now at risk, with estimates suggesting that up to 834,000 people could lose access to Title-X supported services if funding is not restored.^11^ In addition, the Administration’s fiscal year 2026 budget proposal called for eliminating the entire Title X programme, representing a $286 million cut. The elimination of the programme could result in national reductions in contraception access and other preventive services.^12^

While new federal regulations, similar to the 2019 Policy, have not been proposed at the time of publication, such rules could be issued at any time. This paper examines the impact of the 2019 Policy on HIV testing and diagnoses within the Title X programme to inform future discussions on the broad health and equity implications of policies governing the Title X family planning programme. Ensuring a well-functioning Title X programme is not only a matter of public health performance but also vital to protect the fundamental right to accessible, non-discriminatory reproductive and sexual health care.

## Methods

Institutional Review Board approval was not required for this secondary analysis using publicly available data. The unit of analysis for this study is US region (as defined by the US Department of Health and Human Services [HHS], available in Appendix 1).^13^ Primary outcome data for this study are only publicly available by region, requiring a regional-level analysis.

### Variables and data sources

*Primary exposure* The primary exposure variable is exposure to the Policy by HHS region. Exposure is binary, with high exposure regions defined as a net loss of greater than or equal to 25% of the region’s Title X clinics, and low exposure regions defined as a net loss of less than 25% of the region’s Title X clinics from June 2019 to June 2020.^5^ Clinics were considered “lost” if they withdrew from the Title X programme during this period and were considered “added” if they newly joined the Title X programme in this period. This cut-point for high and low exposure to the policy is at the median, where five of the ten regions were coded as high exposure and five were coded as low exposure (Appendix 1). Data were taken from Health and Human Services Office of Population Affairs’ Title X Directories for 2019 and 2020 collated by the Kaiser Family Foundation.^5^

*Primary outcomes* This analysis has two primary outcomes: (1) The number of HIV tests delivered at Title X clinics by region, and (2) The proportion of total HIV cases diagnosed at Title X clinics out of all incident HIV diagnoses by region. Data on the number of HIV tests and diagnoses at Title X clinics were taken from the Title X Family Planning Annual Report National Summaries from 2016 to 2021.^4,7,8,14–16^ This was divided by the total number of regional HIV cases in that year – taken from the Centers for Disease Control (CDC) annual HIV surveillance reports for 2016–2021.^17–22^

### Covariates

Time/year is included as a binary indicator for pre-policy (2016–2018) versus post-policy (2019–2021). Covariates include demographic variables for Title X clients and state-level descriptive variables measured at baseline (2018) aggregated at the region-level. Demographic variables include the proportion of Title X clients by sex (female, male), race (Black, White, and Asian), income (under 101% of federal poverty level, 101%−150%, 151%−200%, and over 201%), and insurance type (uninsured, public, and private) by region. Data on Title X clients come from the Title X Family Planning Annual Report National Summaries 2018.^15^ Region-level descriptive variables, aggregated from state-level data, include: (1) the number of total HIV diagnoses in 2018, from the CDC HIV surveillance 2018 report;^19^ (2) the proportion of states in region that expanded Medicaid eligibility for people up to 138% of the federal poverty level by 2018, from the 2018 Government Accountability Report on Medicaid expansion,^23^ (3) the number of Title X clinics operating by region in 2018, from Title X Family Planning Annual Report 2018 National Summary;^15^ and (4) the average number of days states had a “shelter-in-place” order in effect from January 2020 to April 2021 by region. Days were counted as having a shelter-in-place order in effect if they had any recommendation or requirement to “not leave the house” according to the Oxford COVID-19 Government Response Tracker.^24^ Regional-level covariates were selected to include variables that could influence HIV testing and diagnoses rates at Title X besides the Policy.

### Data analysis

All data were analysed in Stata 16.^25^

#### Baseline analysis

All covariates were tested for their association with exposure at baseline (2018). A two-tailed, two-sample t-test for differences was used to assess the difference in client-demographics and region-level descriptive variables among high exposure and low exposure regions. Only covariates significant at baseline (*p* < .05) were included in modelling given limited power. Likelihood ratio tests were used to assess improved model fit of additional covariates.

#### Mixed effect models

Given the nested nature of our data (regional clusters) with anticipated and known regional variances, we fit mixed linear regression with random intercepts and otherwise fixed regional covariates using maximum likelihood estimation for each outcome (HIV tests per region and proportion of a region’s HIV diagnoses identified at Title X clinics). Models assumed independent covariance with Gaussian errors and observed information matrix standard errors. Regional random intercepts were included with simultaneous estimation on conditional likelihoods. Equations are available in Appendix 2.^13^

A sequential linear mixed-effect modelling approach was used to estimate conditional likelihood on the outcomes. First, we fit null models including random intercepts for regions without covariates. The intraclass correlation coefficient (ICC) was calculated to estimate the proportion of variance attributed to regional random intercepts. Second, we included regional covariates for Policy exposure, pre/post Policy time, and covariates which were significant at baseline including the proportion of Title X clients on public health insurance in 2018, and the proportion of states that expanded Medicaid in 2018. Covariates were the same for both outcome models.

In the final models, an interaction term was added for exposure by pre/post Policy time as the primary difference-in-difference estimator. The interaction term tests if the outcome differs significantly between Policy time periods by exposure group. Improved fit was evaluated using likelihood ratio tests (LRT) to examine if inclusion of additional parameters was justified. Pseudo effect sizes were calculated for the interaction terms by taking the difference between the variance explained by the interaction model compared to the regional covariates model as a proportion of total explained variance in the interaction model.

#### Sensitivity analyses

Authors explored both various model constructions regarding the outcomes and adjustments to the exposure variable. Model selection is discussed here, and results are discussed but not shown below.

Given the limited regional sample size (*n* *=* *10*), single-level ordinary least squares (OLS) regression with 9 region binary indicators was considered, but given problems with collinearity and region exclusions, this approach was dismissed. Single-level OLS with clustered standard errors using the region was also explored. Results were not meaningfully different, and clustered errors can be even more demanding of a large cluster *n*; therefore, our mixed model is preferred.

Primary exposure was also adjusted in sensitivity analysis at a net loss of clinics greater than or equal to 10%, 15% and 30% compared to our original selection of 25%, and an ordinal coding of the four greatest net loss regions coded as 1; the four smallest net loss coded as 0, and the middle two regions coded as .5.

## Results

### Baseline results and descriptive trends

At baseline (2018), high and low exposure regions were similar on demographic and region-level variables, with two exceptions (Table 1). Clinics in high exposure regions served a higher percentage of people on public insurance (41% vs. 30%, *P* = .048) and higher levels of Asian clients (55% vs. 22%, *P* = .007). Among region-level variables, there was a two-tailed, significant difference only in the proportion of states who had expanded Medicaid in high exposure and low exposure regions (97% vs. 58%, *P* = .028).

**Table 1.** Baseline comparison of high and low exposure regions (2018)

	All regions (*n* = 10)	High exposure regions (*n* = 5)	Low exposure regions (*n* = 5)	*P* Value ^a^
**Title X client demographics**				
Female: Avg. %	87	88	87	.681
Race: Avg. %				
Black	20	17	24	.377
White	59	54	64	.185
Asian	3	5	2	.008*
Income level: Avg %				
Under 101%	62	59	64	.309
101%–150%	15	16	14	.145
151%–200%	8	8	7	.298
Over 201%	13	14	12	.486
Insurance status: Avg. %				
Uninsured	39	33	45	.121
Public	35	41	30	.048*
Private	24	24	24	.974
**Regional-level variables**				
Total HIV diagnoses: Regional avg.	3,775	3,188	4,362	.603
% States expanded Medicaid	77	97	58	.028*
Number of Title X clinics: Regional avg.	395	318	473	.314
Days with shelter-in-place orders: Regional average	472	474	471	.914

^a^ Two-sample t-test for difference in means.

*Significant *p* < .05.

**Figure 1.**
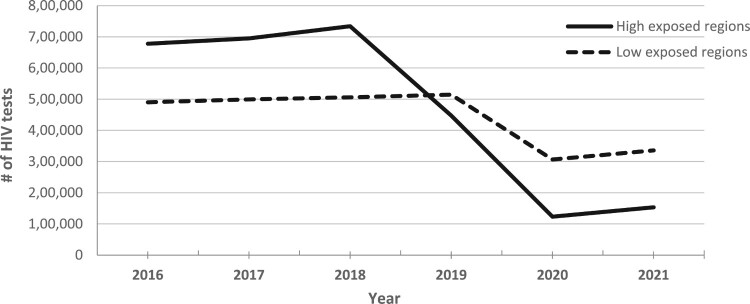
Number of HIV tests provided in high vs. low exposure regions from 2016 to 2021

**Figure 2.**
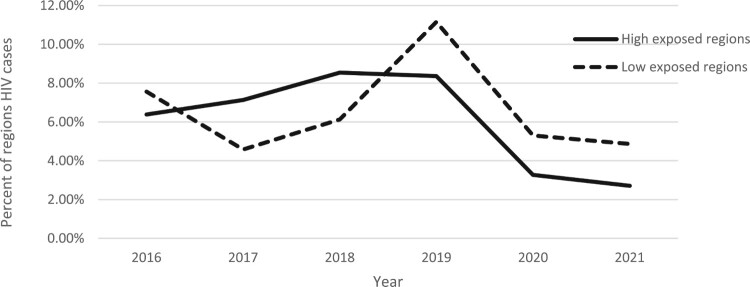
Percent of region’s HIV cases diagnosed at Title X clinics in high vs. low exposure regions from 2016 to 2021

Overall, Title X clinics provided an annual average of 913,903 HIV tests and diagnosed 7.1% of the country’s HIV cases from 2016 to 2021. Prior to the 2019 Policy, high exposure regions conducted an average of 203,759 more HIV tests (Figure 1) and diagnosed an average of 1.3% more of a region’s total HIV cases (Figure 2) every year compared to low exposure regions. This trend reversed in 2019, with high exposure regions conducting 67,242 fewer HIV tests and diagnosing 2.8% less of a region’s HIV cases than low exposure regions. These gaps persisted or widened in 2020, with clinics in high exposure regions conducting 183,411 fewer HIV tests and diagnosing 2.0% less of a region’s HIV cases than in low exposure regions.

### Mixed model results

#### HIV testing at Title X clinics

The null model showed considerable variation in HIV testing between Title X clinics at the regional level (ICC 0.60) indicating that approximately 60% of the total variability in HIV testing is due to differences between regions and justifies the inclusion of region as a random intercept. The regional covariates model showed there was an average decline of 57,346 HIV tests performed at Title X clinics from pre- to post-Policy implementation (Table 2). No other covariates were statistically significant. In comparison to the null model, regional covariates significantly improved fit (LRT *X*^2^ (4) = 24.35, *P* < .001).

**Table 2.** Regional HIV testing at Title X clinics

	Regional covariates model^a^ β (95% CI)	*P* Value	Interaction model^b^ β (95% CI)	*P* Value
Policy exposure: high vs. low	−16,810 (−125,975, 92,354)	.763	18,002 (−92,913, 128,919)	.750
Year: post-policy vs. pre-policy	−57,346 (−79,222, −35,469)	<.000*	−22,533 (−50,299, 5,232)	.112
Proportion of clients on public insurance	524,597 (−30,673, 1,079,832)	.064	524,597 (−30,673, 1,079,832)	.064
Proportion of state Medicaid expansion	−94,551 (−280,598, 91,495)	.319	−94,551 (−280,598, 91,495)	.319
Interaction: Year x Policy exposure			−69,626 (−108,893, −30,359)	<.001*
Pseudo effect-size			−0.24^c^	

^a^ Linear mixed effects model with regional random intercepts.

^b^ Linear mixed effects model with interaction and regional random intercepts.

^c^ Comparing the interaction model to the regional covariates model.

*p < .05.

The interaction models showed that, on average, clinics in high exposure regions conducted 69,626 fewer HIV tests compared to clinics in low exposure regions from pre- to post-Policy periods. This difference was statistically significant (95% CI −108,893 to −30,359 *P* < .001) and improved model fit (LRT *X^2^* (1) = 10.82, *P* < .001). The pseudo effect size for the interaction term was −0.24, suggesting the Policy had a sizeable negative effect on HIV testing at clinics in high exposure regions.

Results from the sensitivity analyses showed the same trends when the exposure variable was re-coded at 10% and 15% or less net loss of sites. While the interaction term was not significant at 30%, both the exposure and the time variable were significant, likely because of auto-regressive effects or sample size effects. The ordinal coded exposure effects were not significant although the patterns were similar.

#### Proportion of regional HIV cases identified at Title X clinics

The null model showed variation in the proportion of HIV cases diagnosed at Title X clinics between regions (ICC 0.31) and justified the inclusion of random intercepts. The regional covariates model showed that overall Title X clinics in high exposure regions identified, on average, 7% less of the region’s total HIV diagnoses compared to low exposure regions (95% CI −10% to −4%; *P* < .001) (Table 3). The coefficient for state Medicaid expansion was also significant, indicating that regions with higher proportions of states which expanded Medicaid had higher proportions of their region’s HIV cases identified at Title X clinics. The inclusion of regional covariates significantly improved fit (LRT *X^2^* (4) = 15.45, *P* = .004).

**Table 3.** Proportion of region’s total HIV cases identified at Title X clinics

	Regional covariates model^a^ β (95% CI)	*P* value	Interaction model^b^ β (95% CI)	*P* value
Policy exposure: high vs. low	−0.07 (−0.10, −0.04)	<.001*	−0.05 (−0.08, −0.02)	<.002*
Year: post-policy vs. pre-policy	−0.01 (−0.29, .01)	.276	0.01 (−0.02, 0.03)	.562
Proportion of clients on public insurance	0.06 (−0.08, 0.20)	.397	0.06 (−0.08, 0.20)	.382
Proportion state Medicaid expansion	0.11 (0.06, 0.15)	<.000*	0.11 (0.06, 0.15)	<.000*
Interaction: Year x Policy exposure			−0.04 (−0.07, 0.00)	.052
Pseudo effect-size			−0.06 ^c^	

^a^ Linear mixed effects model with regional random intercepts.

^b^ Linear mixed effects model with interaction and regional random intercepts.

^c^ Comparing interaction model to regional covariates model.

*p < .05.

The proportion of total HIV diagnoses that occurred in Title X clinics declined, on average, 4% more in high exposure regions compared to low exposure regions between pre- and post-Policy (95% CI −7% to −0%; *P* = .052). A LRT comparing the interaction model to the regional covariates model was non-significant, (*χ*^2^(1) = 3.66, *P* = 0.056). The pseudo effect size for the addition of the interaction term was −0.06, showing a small negative effect of the Policy on the proportion of the region’s HIV diagnoses.

The sensitivity analyses show that when the exposure group threshold is increased to 30% net loss of clinics or when using the ordinal exposure variable, the expansion of Medicaid remains significant, but not at 10% or 15% net loss of sites. While the overall patterns of the estimators remain similar, our exposure variable and interaction term drop below our threshold for significance when the exposure variable is modified.

## Discussion

Our analysis is the first to examine the effect of the 2019 Title X Policy on HIV testing services and diagnoses. We document significant declines in HIV testing and a reduction in the proportion of HIV cases diagnosed at Title X sites as a result of Policy implementation. Our results extend the known negative health impacts of the Policy on contraception provision^26,27^ and STI testing^9^ to include HIV outcomes. Study findings show how policies which target reproductive health providers, such as those in the Title X network, also have implications for HIV service delivery.

Consistent with earlier evaluations examining contraception and STI services, these declines in HIV testing and diagnoses reflect a disruption to the ability of the Title X programme to deliver services under the Policy. In practical terms, clients may still have sought services at the clinics which withdrew from the programme, or they may have sought services at alternative providers. It is not known what proportion of clients stayed, migrated clinics, or forwent services after the 2019 Policy. However, examining the functioning of Title X programme itself remains important, as the programme serves as the major federally funded safety net for reproductive and preventive health services in the US. Evaluating its performance under restrictive policies provides insight into how funding and regulatory changes can weaken the nation’s capacity to deliver equitable, integrated HIV and reproductive health care.

The COVID-19 pandemic, beginning in 2020, coincided with Policy implementation and had known effects on HIV testing and diagnoses in the United States.^28^ HIV diagnoses rates ranged from 13 to 15 cases per 100,000 in the years prior to the pandemic, dropping to 10.9 in 2020 and 12.8 in 2021.^29^ Similarly, we find an overall decline in regional HIV testing from pre- to post-Policy periods – likely related to both the Policy and COVID-19. Our models employ a difference-in-differences approach, which allow us to examine the effect of the Policy independent of temporal trends related to COVID. In our interaction models, we find that the decline in regional HIV testing is significantly greater in high exposure regions than low exposure regions between policy periods. This supports the hypothesis that exposure to the Policy is driving the reduction in regional HIV testing at Title X sites beyond the impact of the COVID-19 pandemic. Additionally, we tested for regional differences in shelter-in-place orders as an indicator of COVID impact, finding that while states may differ, there was no significant difference at the regional level.

Results from our sensitivity analyses suggest that the effects of the Policy on declines in HIV testing are robust to other adjustments of our policy exposure, but this trend does not hold for the proportion of HIV diagnoses. This may be an artefact of unbalanced sampling given our small sample, since otherwise the patterns and trends remain consistent. Compared to the Policy effect size on HIV tests (−0.24), the Policy effect size for the proportion of regional diagnoses was small (−0.06). Regional changes in HIV diagnoses rates are influenced by many factors, including urbanicity, poverty levels, state policy for insurance coverage or protections of sexual minorities, etc.^30–32^ that may have affected HIV diagnoses rates beyond the influence of the Policy. It is therefore expected that the relative influence of the Policy may be smaller, although important, on overall diagnoses rates. Our models also show that Title X clinics detect a higher proportion of HIV cases in regions with more states that have expanded Medicaid. This finding suggests that Title X clinics play a more substantial role in HIV diagnoses in expansion states, likely due to Title X’s role in serving clients on public health insurance. Indeed, Medicaid expansion has been linked to both increased HIV diagnoses rates^33^ and an increase in the proportion of Title X clients with Medicaid.^34^ Together, these data show that Title X clinics remain a vital source of care for both uninsured and Medicaid-insured clients, and that in Medicaid-expansion states, these clinics play a comparatively greater role in HIV case detection – likely due to increased access and not greater need. Medicaid expansion may be an important avenue to increase reimbursements and support financial stability at Title X clinics.

This analysis only examined the impact of the Policy on HIV testing and diagnoses by the Title X programme. However, it is possible that the Policy had broader client impacts on HIV services at the state level, as clinics that left the programme suffered funding cuts or closure. Indeed, evidence suggests that some Title X clinics that left the programme and lost federal funding reported service reductions, staff shortages, or closures due to Policy-related funding cuts.^27,35–38^ These financial difficulties had a known impact on client numbers and contraception provision^27,38^ and could have similarly impacted HIV service delivery. Full clinic closures may have been especially disruptive for a client base which relied on a Title X provider for HIV testing and faced additional barriers getting care at other locations.

The US goal to eliminate HIV as a public health threat by 2030^39^ will only be achieved through policies that promote HIV testing and diagnoses. The Title X programme is a key contributor to the HIV landscape in the US, diagnosing between 4% and 10% of the total HIV cases from 2016 to 2021. Strong funding for the Title X programme is likely to carry HIV benefits, especially for low-income people who make up 65% of the Title X programme’s client base.^40^ Supporting the programme is therefore not only a matter of epidemiologic impact, but also of health equity and the right to access essential preventive services. Policies that remove restrictions and enable Title X funds to support a full range of qualified sexual and reproductive health providers are critical to reducing inequities in HIV outcomes and ensuring that all communities can access high-quality care. Conversely, any future policies akin to the 2019 Policy discussed in the paper will have predictably negative effects on HIV testing and diagnosis rates in the Title X programme. US policymakers have an obligation to consider both the public health consequences and the equity implications of restricting Title X funding, particularly given the programme’s role in upholding access to essential HIV and other health services for vulnerable populations.

### Limitations

Outcome data were only available at the regional level, which does not allow for state-level analyses and limits statistical power. While we considered other single-level models using regional indicators or regional clustered standard errors, our mixed model appears the most robust. Despite limited power, statistically significant results were found, suggesting there was a significant policy influence at the regional level. Additionally, no sex-disaggregated Title X data were available for either HIV testing or diagnoses. These data would be important to examine if the Policy had disproportionate effects based on sex. In this non-randomised study design, we examined several potential confounding factors that differed between high and low exposed regions, including the impact of COVID-19-related restrictions. However, other non-measured confounders related to the pandemic may exist. Lastly, results of this analysis can only be interpreted as the policy’s effect on HIV testing and diagnoses in the Title X programme, not as the effect of the policy on the broader HIV landscape in the US. It is not known if the drop in HIV testing and diagnoses at Title X clinics is indicative of an overall drop or a switch to other non-Title X testing mechanisms.

## Conclusion

The 2019 Policy on Title X prompted widespread clinic withdrawal and diverted funding away from established HIV testing sites in the US. As a result, the programme suffered marked declines in HIV testing and diagnoses. Sound health policy that advances efforts to end the US HIV epidemic will support reproductive health clinics, such as those in the Title X programme, as key providers of HIV services. Conversely, policies that endanger the Title X network also threaten to weaken the US HIV response. Future Title X policy decisions should avoid repeating the 2019 restrictions, which harmed access to sexual and reproductive health care and weakened the programme’s HIV testing and diagnosis efforts.

## Supplementary Material

Appendices

## Data sharing

All data used in this analysis are publicly available. Collated data may be shared upon request to the corresponding author.

## Provenance statement

This article was not commissioned and went through external peer review.
