# Supplementary material for: Declines in HIV testing and diagnoses: a policy analysis of the 2019 Title X federal regulations on family planning clinics
Source: Sex Reprod Health Matters. 2026 May 8;33(1):2670146. doi: 10.1080/26410397.2026.2670146 (PMC13248498; doi:10.1080/26410397.2026.2670146)
Supplement: Appendices [file ZRHM_A_2670146_SM9701.docx]

**Declines in HIV Testing and Diagnoses: A Policy Analysis of the 2019 Title X Federal Regulations on Family Planning Clinics**

**Appendix 1.**

| **Regional designation and changes in Title X site participation** | | | |
| --- | --- | --- | --- |
| HHS Region^ | States | Pre- to post-policy net clinic change | Exposure to Policy* |
| 1 | Connecticut, Maine, Massachusetts, New Hampshire, Rhode Island, Vermont | -70% | High |
| 2 | New Jersey, New York, Puerto Rico and the U.S. Virgin Islands | -73% | High |
| 3 | Delaware, District of Columbia, Maryland, Pennsylvania, Virginia, West Virginia | -14% | Low |
| 4 | Alabama, Florida, Georgia, Kentucky, Mississippi, North Carolina, South Carolina, Tennessee | 1% | Low |
| 5 | Illinois, Indiana, Michigan, Minnesota, Ohio, Wisconsin | -39% | High |
| 6 | Arkansas, Louisiana, New Mexico, Oklahoma, Texas | 7% | Low |
| 7 | Iowa, Kansas, Missouri, Nebraska | -9% | Low |
| 8 | Colorado, Montana, North Dakota, South Dakota, Utah, Wyoming | -16% | Low |
| 9 | Arizona, California, Hawaii, Nevada, American Samoa, Commonwealth of the Northern Mariana Islands, Federated States of Micronesia, Guam, Marshall Islands, Republic of Palau | -27% | High |
| 10 | Alaska, Idaho, Oregon, Washington | -67% | High |
| ^ U.S. regions as defined by the Department of Health and Human Services (HHS)  **A high exposure designation indicates any region with more than 25% net clinic loss* | | | |

**Appendix 2.** **Model notation and beta-descriptions**

Model outcomes include; 1) HIV tests per region (*hiv_test*) and, 2) Proportion of a region’s HIV diagnoses identified at Title X clinics (*prop_pos*).

**Null models**

The formulations for these null models were as follows where β0_j_ represents the random intercept for each region and ε_ij_ is the residual error.

**hiv_testij=β0j+ εij**

**prop_posij= β0j+ εij**

**Regional covariate models**

The regional covariates models included covariates for Policy exposure (*exposed*), pre/post Policy time periods (*year_bi*), proportion of Title X clients on public health insurance in the region in 2018 (*public*), and the proportion of states that expanded Medicaid in 2018 (*expand*). The regional covariates models are given by the following where β0_j_ represents the random intercept for each region, β1 represents the average change in the outcome in exposed vs. unexposed regions, β2 represents the average change in the outcome post-policy vs. pre-policy, β3 represents the average change in the outcome per unit increase in the proportion of Title X clients on public health insurance served, β4 represents the average change in the outcome per unit increase in the proportion of states that expanded Medicaid, and ε_ij_ is the residual error.

**hiv_testij=β0j+ β1exposedij+ β2 year_biij+ β3publicij+ β4expandij+ εij**

**prop_posij= β0j+ β1exposedij+ β2 year_biij+ β3publicij+ β4expandij+ εij**

**Interaction models**

In the final models, an interaction term was created for exposure and pre/post Policy time (*exposed x year_bi*) and added to the regional covariates models. The interaction models are as follows where β5 is the additive average difference in the outcome for the combined effect of exposed regions and post Policy implementation.

**hiv_testij= β0j+ β1exposedij+ β2 year_biij+ β3publicij+ β4expandij+β5expandij x year_biij+ εij**

**prop_posij= β0j+ β1exposedij+ β2 yearbiij+ β3publicij+ β4expandij+β5expandij x year_biij+ εij**
